# NcPuf1 Is a Key Virulence Factor in *Neospora caninum*

**DOI:** 10.3390/pathogens9121019

**Published:** 2020-12-02

**Authors:** Chenrong Wang, Congshan Yang, Jing Liu, Qun Liu

**Affiliations:** National Animal Protozoa Laboratory, College of Veterinary Medicine, China Agricultural University, Beijing 100193, China; wangchenrong222@sina.com (C.W.); congshanyang@sina.cn (C.Y.)

**Keywords:** *Neospora caninum*, Puf protein, tachyzoites replication, cyst formation

## Abstract

Background: *Neospora caninum* is an apicomplexan parasite that infects many mammals and particularly causes abortion in cattle. The key factors in its wide distribution are its virulence and ability to transform between tachyzoite and bradyzoite forms. However, the factors are not well understood. Although Puf protein (named after Pumilio from *Drosophila melanogaster* and fem-3 binding factor from *Caenorhabditis elegans*) have a functionally conserved role in promoting proliferation and inhibiting differentiation in many eukaryotes, the function of the Puf proteins in *N. caninum* is poorly understood. Methods: The CRISPR/CAS9 system was used to identify and study the function of the Puf protein in *N. caninum*. Results: We showed that *N. caninum* encodes a Puf protein, which was designated NcPuf1. NcPuf1 is found in the cytoplasm in intracellular parasites and in processing bodies (P-bodies), which are reported for the first time in *N. caninum* in extracellular parasites. NcPuf1 is not needed for the formation of P-bodies in *N. caninum*. The deletion of NcPuf1 (ΔNcPuf1) does not affect the differentiation in vitro and tissue cysts formation in the mouse brain. However, ΔNcPuf1 resulted in decreases in the proliferative capacity of *N. caninum* in vitro and virulence in mice. Conclusions: Altogether, the disruption of NcPuf1 does not affect bradyzoites differentiation, but seriously impairs tachyzoite proliferation in vitro and virulence in mice. These results can provide a theoretical basis for the development of attenuated vaccines to prevent the infection of *N. caninum*.

## 1. Introduction

*Neospora caninum* is an apicomplexan protozoan parasite that is closely related to *Toxoplasma gondii*. However, *N. caninum* does not infect humans [1]. *N. caninum* is the primary pathogen causing an abortion in cattle and brings enormous economic losses to the cattle industry around the world [2,3,4].

Similar to *T. gondii*, *N. caninum* tachyzoites can differentiate into bradyzoites that cause chronic infections, which is asymptomatic in immunocompetent hosts. However, upon host immunosuppression, *N. caninum* switches from its bradyzoite form to its fast-replicating tachyzoites form, resulting in severe tissue damage and abortion [5]. Bradyzoites in tissue cysts cannot be eliminated by anti-parasitic drugs or vaccines that can limit the proliferation of tachyzoites. Therefore, understanding the factors that regulate the virulence of *N. caninum* and the molecular mechanism of the conversion between tachyzoites and bradyzoites are critical for identifying new molecular targets for the treatment of neosporosis.

Most eukaryotic organisms rely on tight control of gene expression to complete their life cycle. The expression of many genes is altered in the tachyzoite-to-bradyzoite differentiation [6,7]. The post-transcriptional regulation of genes is more rapid than that at the transcriptional, which is considered a powerful growth strategy in various environments. In eukaryotic cells, RNA-binding protein (RBP) is vital for posttranscriptional gene regulation by RNA metabolism [8]. Puf (named after Pumilio in *Drosophila melanogaster* and fem-3 binding factor in *Caenorhabditis elegans*) protein is an RBP [9,10]. Some Puf proteins can be recruited into ribonucleoprotein (RNP) granules to control target mRNA’s fate under stress. RNP granules include stress granules (SGs) and processing bodies (P-bodies). However, the functions of these two RNPs are different. SGs can inhibit the translation of the targeted mRNAs while P-bodies degrade the targeted mRNAs. Poly(A)-mRNA-binding protein (PABP) was only found in SGs while decapping enzymes (Dcps) were only detected in P-bodies, so we can distinguish these two structures by PABP and Dcps. Although Puf proteins have various functions, they play a functionally conserved role in promoting proliferation and inhibiting differentiation [9]. For example, the deletion of PfPuf2 (ΔPfPuf2) or PbPuf2 (ΔPbPuf2) promotes the differentiation of gametocytes in *Plasmodium* [11,12,13]. Although Puf proteins have been studied in many eukaryotes, their function in *N. caninum* is poorly understood.

Here, we showed that *N. caninum* encodes a Puf protein named NcPuf1. NcPuf1 is found in the cytoplasm in intracellular parasites and in P-bodies, which are reported for the first time in *N. caninum* in extracellular parasites. The deletion of NcPuf1(ΔNcPuf1) does not affect the formation of P-bodies in *N. caninum* and the differentiation in vitro and tissue cysts formation in the mouse brain. However, ΔNcPuf1 resulted in decreases in the proliferative capacity of *N. caninum* in vitro and virulence in mice.

## 2. Materials and Methods

### 2.1. Cell and Parasite Culture

Human foreskin fibroblast cells (HFFs, ATCC^®^ SCRC-1041™, Manassas, VA, USA) were cultured in DMEM with 25 mM glucose and 4 mM glutamine, supplemented with 10% fetal bovine serum (FBS, Gibco, Rockville, MD, USA). *N. caninum* parasites were maintained in HFFs by serial passages, as described previously [14]. Both cells and parasites were incubated at 37 °C with 5% CO_2_ in a humidified incubator.

### 2.2. Construction of Transgenic Parasite Strains

To identify the expression of NcPuf1 in *N. caninum*, we used the CRISPR/CAS9 system to add a HA epitope at the C-terminus of NcPuf1. The primer sequences used in this study are listed in Supplementary Table S1. pSAG1::CAS9-NcU6::sgNcPuf1^1^ was constructed as previously described [14,15]. In brief, we used the pSAG1::CAS9-NcU6::sgNcUPRT stored in our laboratory as a template to amplify the cas9 (P1-2 primers), NcU6 (P3-4), and the backbone fragment (P5-6) containing the NcPuf1 gRNA sequence (5′-GCGGCGGGAGGGTTGAACGA-3′). The cas9, NcU6, and the backbone fragment were ligated with ClonExpress MultiS One Step Cloning Kit (Vazyme, Nanjing, China). To obtain homologous recombination fragments, upstream (1147 bp) (P7-8) and downstream (1169 bp) region (P9-10) of the gNcPuf1^1^ sequence were amplified, the backbones (pLIC and HA-DHFR) (P11-14) were amplified from pLIC-HA-DHFR-NcGRA17 stored in our laboratory, then upstream and downstream region and the backbones were ligated to construct the plasmids (pLIC-HA-DHFR-NcPuf1). Nc-Liv was co-transfected with the pSAG1::CAS9-NcU6::sgNcPuf1^1^ and the linearized pLIC-HA-DHFR-NcPuf1 (P15-16) that was obtained from the pLIC-HA-DHFR-NcPuf1. The NcPuf1-HA-tagged strains were screened using pyrimethamine (1 μM). The selected NcPuf1-HA-tagged strains were identified using PCR (P17-20), western blotting, and immunofluorescence assay (IFA).

The NcDcp-FLAG tagged strain was constructed to detect the expression of NcDcp1 in *N. caninum*. We used the pSAG1::CAS9-NcU6::sgNcUPRT as a template to amplify the cas9 (P1-2), the backbone(P6, P21), and NcU6 fragment (P3, P22) containing the NcDcp1 gRNA sequence (5′-AAAGAGAAACGACCGGTCGC-3′). The cas9, NcU6, and the backbone fragment were ligated to construct the plasmids (pSAG1::CAS9-NcU6::sgNcDcp1). Upstream (P23-P24) (677 bp) and downstream (P25-P26) (777 bp) regions of the gNcDcp1 sequence were amplified, the backbones (pLIC and FLAG-CAT)(P27-P30) were amplified from pLIC-FLAG-CAT stored in our laboratory, then upstream and downstream region and the backbones were ligated to construct the plasmids (pLIC-FLAG-CAT-NcDcp1). The NcPuf1-HA-tagged strain was co-transfected with the pSAG1::CAS9-NcU6::sgNcDcp1 and the linearized pLIC-FLAG-CAT-NcDcp1(P15-P16). The NcDcp1-FLAG-tagged strains were screened using chloramphenicol (20 μM). The selected NcDcp1-FLAG-tagged strains were identified using PCR (P31-P34), western blotting, and IFA.

pSAG1::CAS9-NcU6::sgNcPuf1^2^ for disrupting the NcPuf1 gene was constructed by replacing the gRNA of NcUPRT in the above plasmid with the gNcPuf1^2^ sequence(5′-GTGCGCTAAGGGCATCGGGT-3′)(P1-P2, P3, P6, P35, P36). 5’flanking (P37-P38) (923 bp) and 3’flanking sequences (P39-P40) (857 bp) of the NcPuf1 gene were amplified, the backbones (PTCR and CAT-RFP) (P41-P44) were amplified from pTCR-CAT-RFP stored in our laboratory, then 5’ and 3’flanking sequence and the backbones were ligated to construct the plasmid (pTCR-NcPuf1-CAT-RFP). The parental Nc-Liv strain was co-transfected with pSAG1::CAS9-NcU6::sgNcPuf1^2^ and the linearized pTCR-NcPuf1-CAT-RFP (P45-P46). The parasites were screened using chloramphenicol (20 μM). The selected NcPuf1-deficient clones obtained by limiting dilution method, designated ΔNcPuf1, were identified by PCR (P47-P54), quantitative real-time PCR (qRT-PCR) (P55-P58), and red fluorescent signal.

To obtain the NcPuf1 complementary strain, the NcPuf1 gene was amplified, the backbones (pNcUPRT and HA-DHFR) (P59-P62) were amplified from pNcUPRT::DHFR-NcGRA17 stored in our laboratory, then the NcPuf1 gene (P63-P64) and the backbones were ligated to construct the plasmids (pNcUPRT::DHFR-NcPuf1). ΔNcPuf1 strain was co-transfected with pSAG1::CAS9-NcU6::sgNcUPRT and the linearized pNcUPRT::DHFR-NcPuf1(P15-16) to complement NcPuf1 with an HA-tag in ΔNcPuf1 strain by targeting the NcUPRT gene. Then, the parasites were selected with pyrimethamine (1 μM). The selected NcPuf1 complementary clones, designated iΔNcPuf1, were confirmed via PCR (P65-P70), western blotting assay, and IFA.

We construct the NcDcp-HA tagged strains to analyze the localization of NcDcp1 in the ΔNcPuf1 strain. Upstream (P71-P72) (639 bp) and downstream (P73-P74) (739 bp) regions of the gNcDcp1 sequence were amplified, the backbones (pLIC and HA-DHFR) (P75-P78) were amplified, then upstream, downstream region and the backbones were ligated to construct the plasmid (pLIC-HA-DHFR-NcDcp1). The ΔNcPuf1 strain was co-transfected with the pSAG1::CAS9-NcU6::sgNcDcp1 and the linearized pLIC-HA-DHFR-NcDcp1 (P15-16). The NcDcp1-HA-tagged strains were screened using pyrimethamine (1 μM). The selected NcDcp1-HA-tagged strains were identified using PCR (P31, P79, P34, P80), western blotting, and IFA.

### 2.3. Western Blotting Assay and Immunofluorescence Assays

Western blotting assay and immunofluorescence assays were performed as previously described [14].To obtain *N. caninum* tachyzoites, we scraped the parasites and the cells with a scraper and passed through a 27-gauge needle 8 times, and then the parasites were passed through a 5.0 μm filter and centrifuged. The obtained parasites were lysed with RIPA buffer (Solarbio, Beijing, China). Then, the lysates were run in 12.5% SDS-PAGE and transferred to a polyvinylidene fluoride membrane. Then, it was blocked with 5% skim milk for 1 h at room temperature and incubated with primary antibodies used in this study were rabbit anti-FLAG (Sigma-Aldrich, Saint Louis, MO, USA; 1:500), mouse anti-HA (Sigma-Aldrich, Saint Louis, MO, USA; 1:500), or rabbit anti-*N. caninum* F-actin subunit beta (Ncactin, National Animal Protozoa Laboratory, China Agricultural University) (1:5000) and HRP-labeled secondary antibody. Finally, the reactive bands were visualized using enhanced chemiluminescence reagents (Huaxinbio, Beijing, China).

For IFA, HFF cells were cultured on a glass slide in a 12-well plate, 1 × 10^5^ parasites per well were added into 12-well plate and cultured at 37 °C with 5% CO_2_ for 28 h, then 4% paraformaldehyde was added to fix for 30 min at room temperature before being permeabilized with 0.25% TritonX-100. Subsequently, parasite-infected cells were blocked with 3% BSA at 37 °C for 1 h and incubated with primary antibodies antibodies that used in this study were mouse anti-FLAG (Sigma-Aldrich, Saint Louis, MO, USA; 1:100), rabbit anti-FLAG (Sigma-Aldrich, Saint Louis, MO, USA; 1:100), mouse anti-HA (Sigma-Aldrich, Saint Louis, MO, USA; 1:100), rabbit anti-human PABP with 60.59% sequence identity with *N. caninum* (Proteintech, Wuhan, China; 1:100), rabbit anti-HA (Sigma-Aldrich, Saint Louis, MO, USA; 1:100), FITC-labeled Dolichos biflorus agglutinin (DBA-FITC) (1:100), or a rabbit anti-NcSRS2 antibody (National Animal Protozoa Laboratory, China Agricultural University) (1:250) and secondary antibodies. To analyze the localization of NcPuf1 in extracellular parasites, we collected freshly purified parasites, and then added the parasite on the glass slide. The procedure of IFA was the same as described above.

### 2.4. Quantitative Real-Time PCR

The purified parasites (Nc-Liv and ΔNcPuf1) were obtained as described above. Then, total RNA was extracted and reverse transcribed into cDNA as described previously [16,17]. The transcription levels of NcPuf1 (P49-P50) was checked by qRT-PCR using the SYBR Green Master Mix (Vazyme Biotech, Nanjing, China) on the Roche LightCycler 480 Real-Time PCR System. The Ncactin (P47-P48) was used as an internal reference. The experiments were repeated three times independently, each with three technical replicates.

### 2.5. Plaque Assay and Parasite Intracellular Replication Assay

The plaque assay was performed as previously described [14,16]. In brief, three hundred freshly purified parasites were seeded into HFF cells, followed by incubation at 37 °C with 5% CO_2_ for 9 d. Then, the plates were fixed and stained with 2% crystal violet. These samples were washed with PBS. Plaque size was measured via the Pixel plugin using Adobe Photoshop CC2018 software (Adobe, San Jose, CA, USA). One of three independent experiments is presented.

The parasite intracellular replication assay was performed as previously described [14,16]. HFF cells grown on 12-well plates were infected with 5 × 10^5^ parasites per well. The unbound parasites were removed by washing with PBS after 1 h, followed by incubation again for 30 h. Then, the replication ability of the parasites was observed by IFA using rabbit anti-NcSRS2 antibody. The numbers of parasites per vacuole were determined by counting 100 parasitophorous vacuoles under a fluorescence microscope (Olympus Co., Tokyo, Japan). The data were obtained from three independent experiments.

### 2.6. Bradyzoite Differentiation Assay In Vitro

1 × 10^4^ parasites were seeded into HFF cells in 12-well plates with a culture medium. We replaced the culture medium with pH 8.2 medium after 6 h, followed by incubation again for 4 d under differentiation conditions (pH 8.2, low CO_2_). Then, the samples were performed as described for the immunofluorescence assay. Cyst was detected by IFA using FITC-labeled Dolichos biflorus agglutinin (DBA-FITC) and rabbit anti-NcSRS2 antibody [18]. Numbers of the cyst were determined by counting 100 randomly selected parasitophorous vacuole. The experiments were repeated three times independently.

### 2.7. Animal Infection Experiments

For the parasite virulence assay, BALB/c mice (5 weeks old) were raised in sterile cages in a barrier environment and had free access to clean water and food. 2 × 10^6^ tachyzoites were used to infect mice (five mice per group) by intraperitoneal injection (i.p.) as previously described [14,16]. All mice were observed for 45 days. They were monitored every day for their clinical signs and mortality. If the mouse could not eat or drink for more than 24 h or lose 20% of its normal body weight, we would humanely euthanize the mouse by cervical dislocation.

For the brain cyst assay, the Nc-Liv strain produced few cysts in the BALB/c mouse brain, while more cysts were detected in Kunming mice. Thus, female Kunming mice (7 weeks old) were raised in sterile cages in a barrier environment and had free access to clean water and food. 4 × 10^6^ tachyzoites were used to infect female Kunming mice (nine mice per group) by i.p. All mice were observed for 3 months. The mice were monitored and humanely euthanized as described above. The brains were homogenized in PBS. Then, the brain homogenate was incubated with FITC-tagged DBA for 30 min. Brain cyst counts were determined by DBA-FITC staining.

All animal experiments were approved by the Institutional Animal Care and Use Committee of China Agricultural University (Approval No.: 18049).

### 2.8. Statistical Analysis

The statistical analyses were performed by GraphPad Prism 5 (GraphPad Software Inc., La Jolla, CA, USA). Data were analyzed using the two-tailed, unpaired Student’s *t*-test or one-way ANOVA or Chi-square test or Log-rank test. *p*-values are represented by asterisks: * 0.01 < *p* < 0.05; ** 0.001 < *p* < 0.01; *** *p* < 0.001.

## 3. Results

### 3.1. NcPuf1 Protein Is Expressed in N. caninum

A BLASTP search of the *N. caninum* genome in the ToxoDB database (http://toxodb.org) using the Puf domain of *T. gondii* identified two putative Puf genes (NCLIV_026460 and NCLIV_011170). The NCLIV_026460 gene encoding of 1537 amino acids shares 78.71% similarity with TgPuf1, the NCLIV_011170 gene encoding of 2011 amino acids shares 57.13% similarity with TgPuf2. Based on their homology degrees to the *T. gondii* Pufs, the NCLIV_026460 and NCLIV_011170 genes were named NcPuf1 and NcPuf2, respectively. NcPuf1 protein shares 25.83% similarity with NcPuf2. The homology of them is limited to the Puf domain.

**Figure 1:**
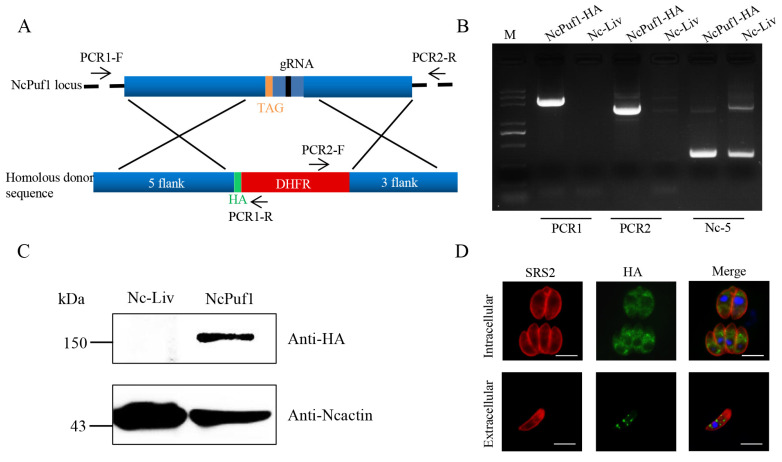
Identification and localization of NcPuf1 protein. (**A**) Schematic representation of adding an HA epitope at the C-terminus of NcPuf1 using the CRISPR/CAS9 system. PCR1/ PCR2-F/R represent primers. These primers are used to identify the recombination of the NcPuf1-HA strain. (**B**) The NcPuf1-HA-tagged strains were identified using PCR. PCR1 and PCR2 were used to identify the 5′ and 3′ insertions of HA-DHFR, respectively. The Nc-5 gene is a positive control gene. (**C**) Western blotting showed NcPuf1 was expressed in *N. caninum*. (**D**) HA-tagged NcPuf1 was located in the cytoplasm in intracellular parasites and presented a granular cytoplasmic staining pattern in extracellular parasites. Bars, 5 μm.

Next, to identify the expression and localization of the NcPuf1 in *N. caninum*, we tagged the NcPuf1 with HA tag in *N. caninum*. The parental Nc-Liv strain was cotransfected with the pSAG1::CAS9-NcU6::sgNcPuf11 and linearized pLIC-HA-DHFR-NcPuf1 (Figure 1A). Then, the parasites were screened using pyrimethamine. The transfected parasites were recombined correctly by PCR (Figure 1B). We detected a protein band by western blot, consistent with the predicted sizes of the NcPuf1-HA (Figure 1C). As expected for a conserved function of Pufs in translational regulation, the NcPuf1 protein was located in the cytoplasm in intracellular parasites (Figure 1D). When parasites were released from host cells, the NcPuf1 protein showed a granular cytoplasmic staining pattern in the cytoplasm (Figure 1D). Overall, these results demonstrated that NcPuf1 protein was expressed in *N. caninum*.

### 3.2. NcPuf1 Is Recruited into Processing Bodies in Extracellular Parasites

It has been previously described that RNP granules of *T. gondii* are present in extracellular parasites [19]. Therefore, we speculated that the observed punctate cytoplasmic structures might be RNP granules. The RNP granules likely control the fate of these mRNAs. RNP granules usually include SGs and P-bodies. SGs contain many repressed mRNAs and RBPs, while P-bodies have many mRNA decay factors and RBPs. To verify whether NcPuf1 is located in SGs, we examined its colocalization with PABP, and found that they did not colocalize (Supplementary Figure S1). Therefore, we surmised that NcPuf1 might be located in P-bodies, but P-bodies have not previously been reported in *N. caninum*. Decapping enzymes (Dcps) are evolutionarily conserved markers of P-bodies in most eukaryotic organisms [20,21]. A BLASTP search of the *N. caninum* genome in the ToxoDB database identified a putative Dcp gene (NCLIV_003520) encoding of 577 amino acids. It shares 23.40% and 22.43% similarity with *Cryptosporidium hominis* and *Plasmodium vivax* Dcp1, respectively. So we named it NcDcp1 (decapping enzyme 1).

**Figure 2:**
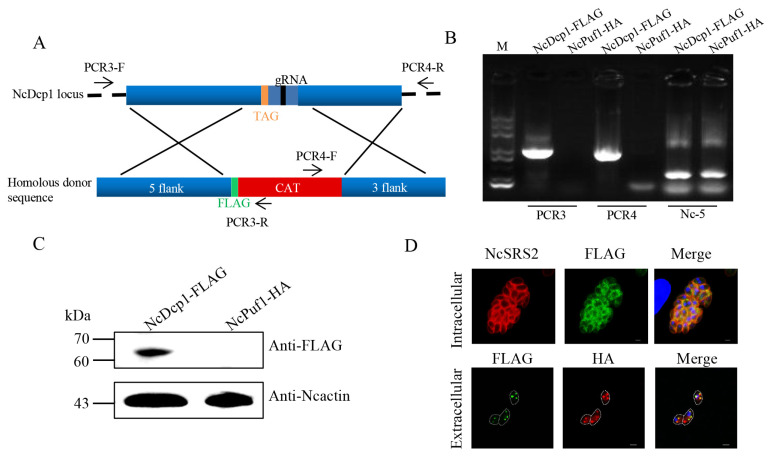
The subcellular localization of NcPuf1 in extracellular parasites. (**A**) Schematic representation of adding a FLAG epitope at the C-terminus of NcDcp1 in the parental NcPuf1-HA strain. PCR3/PCR4-F/R represent primers used to identify the recombination of the NcDcp1-FLAG-tagged strains. (**B**) The NcDcp1-HA-tagged strains were identified using PCR. PCR1 and PCR2 were used to identify the 5′ and 3′ insertions of FLAG-CAT, respectively. The Nc-5 gene is a positive control gene. (**C**) Western blot analysis showed that *N. caninum* expressed decapping enzyme 1 (Dcp1), a conserved marker of P-bodies. (**D**) The HA-tagged NcPuf1 protein colocalized with the FLAG-tagged NcDcp1 protein in extracellular parasites. IFA analysis showed that the FLAG-tagged NcDcp1 protein presented a granular cytoplasmic staining pattern in intracellular parasites and colocalized with the HA-tagged NcDcp1 protein in extracellular parasites. Bars, 2 μm.

To identify the expression and localization of the NcDcp1 in *N. caninum*, we tagged the NcDcp1 with FLAG tag in the NcPuf1-HA parasite strain. The parental NcPuf1-HA parasite strain was cotransfected with the pSAG1::CAS9-NcU6:: sgNcDcp1 and linearized pLIC-FLAG-DHFR- NcDcp1 to generate a NcPuf1-HA-NcDcp1-FLAG parasite strain (Figure 2A). The transfected parasites were recombined correctly by PCR (Figure 2B). Western blotting using rabbit anti-FLAG antibody was used to confirm that *N. caninum* expressed the NcDcp1 (Figure 2C). IFA using mouse anti-FLAG antibody showed that the NcDcp1 protein presented a granular cytoplasmic staining pattern in intracellular parasites (Figure 2D), which was consistent with the characteristics of P-bodies. Then, we found that the NcPuf1 proteins colocalized with NcDcp1 in extracellular parasites by IFA using rabbit anti-FLAG and mouse anti-HA antibody (Figure 2D). Together, the results showed that NcPuf1 was recruited into P-bodies in extracellular parasites.

### 3.3. NcPuf1 Knockout and Complementary Strains

**Figure 3:**
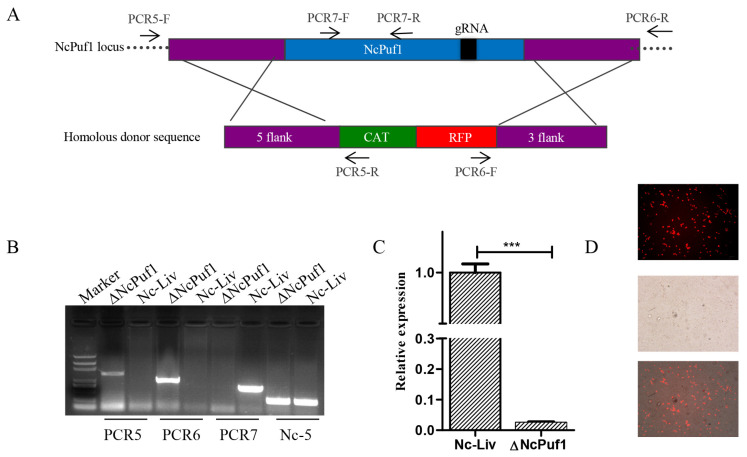
Identification of the ∆NcPuf1 strain. (**A**) Schematic representation of deleting NcPuf1 gene. PCR5/PCR6-F/R represent primers used to identify the recombination of the ∆NcPuf1 strains. PCR7-F and PCR7-R were used to identify the deletion of the NcPuf1 gene. (**B**) The deletion of the NcPuf1 gene in the Nc-Liv strain was confirmed by PCR. The Nc-5 gene is a positive control gene. (**C**) The deletion of the NcPuf1 gene in the Nc-Liv strain was further confirmed via qRT-PCR. The Ncactin was used as an internal reference. *** *p* < 0.001. (**D**) The ∆NcPuf1 strain was further evaluated using an inverted fluorescence microscope. The ∆NcPuf1 strain could show red fluorescence.

To study the role of NcPuf1 in tachyzoites, we constructed a NcPuf1 knockout strain. The parental Nc-Liv strain was co-transfected with pSAG1::CAS9-NcU6::sgNcPuf12 and the linearized pTCR-NcPuf1-CAT-RFP to replace the NcPuf1 gene with the chloramphenicol resistance gene (CmR) and red fluorescence protein gene (RFP) (Figure 3A). Following CmR selection, we isolated NcPuf1 knockout clones. ΔNcPuf1 was confirmed via PCR and quantitative qRT-PCR analysis (Figure 3B,C). In addition, RFP was expressed in the ΔNcPuf1 strains (Figure 3D), further supporting the deletion of NcPuf1.

**Figure 4:**
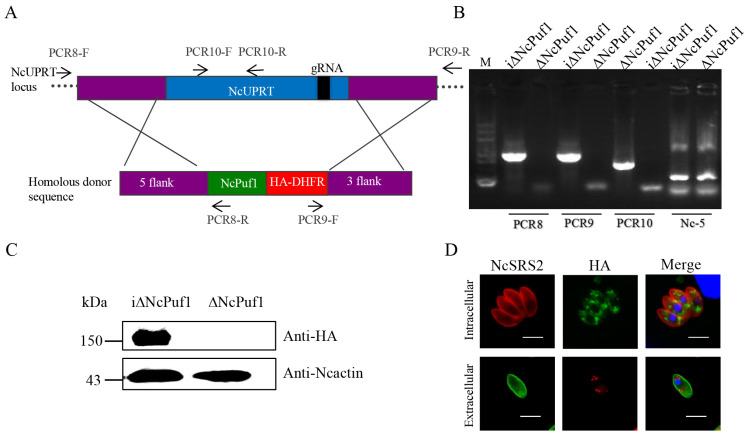
Identification of the i∆NcPuf1 strain. (**A**) Schematic representation of the complement of NcPuf1 gene. PCR8/PCR9-F/R represent primers used to identify the recombination of the ∆NcPuf1 strains. PCR10-F and PCR10-R were used to identify the deletion of the NcUPRT gene in the i∆NcPuf1 strain. (**B**) T the complement of NcPuf1 gene in the ∆NcPuf1 strain was confirmed by PCR. The Nc-5 gene is a positive control gene. (**C**) Western blotting showed NcPuf1 was expressed in the i∆NcPuf1 strain. (**D**) The NcPuf1 gene was successfully complemented by IFA. The NcPuf1 gene in the i∆NcPuf1 strain was located in the cytoplasm in intracellular parasites and presented a granular cytoplasmic staining pattern in extracellular parasites. Bars, 5 μm.

Then we constructed the NcPuf1 complementary strain (iΔNcPuf1) (Figure 4A). ΔNcPuf1 strain was co-transfected with pSAG1::CAS9-NcU6::sgNcUPRT and the linearized pNcUPRT::DHFR-NcPuf1 to complement NcPuf1 with an HA-tag in ΔNcPuf1 strain by targeting the NcUPRT gene. Then, the parasites were selected with pyrimethamine. The iΔNcPuf1 clones were confirmed via PCR (Figure 4B). NcPuf1 was expressed in iΔNcPuf1 strains via western blotting assay and IFA using mouse anti-HA antibody (Figure 4C,D).

### 3.4. NcPuf1 Is Vital for Parasite Growth In Vitro and Virulence in Mice

**Figure 5:**
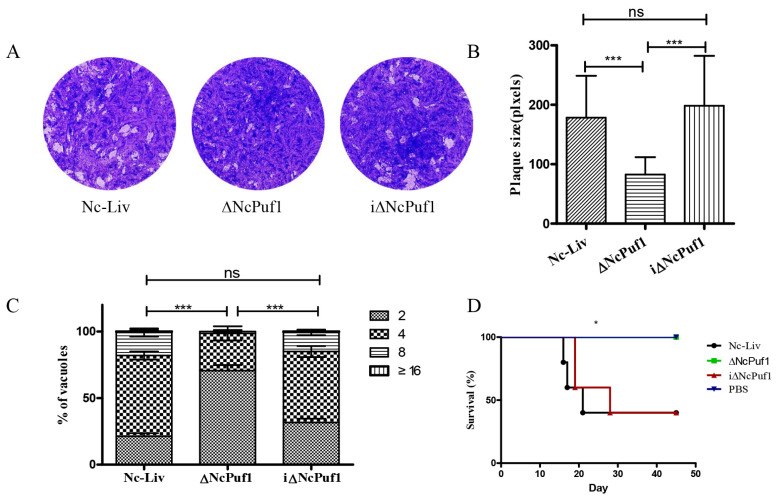
Deletion of NcPuf1 affects parasite replication and virulence. (**A**) Plaque assay of the Nc-Liv, ΔNcPuf1 and iΔNcPuf1 strains. (**B**) Plaque size was measured with the Adobe Photoshop CC2018 (Adobe, San Jose, CA, USA) using the Pixel plugin. Data are analyzed with One-way ANOVA with Tukey. (**C**) Intracellular replication of the ∆NcNcPuf1, Nc-Liv and i∆NcNcPuf1 strains. Data are analyzed with Chi-square test. (**D**) Mouse survival after infection with different strains (Nc-Liv, ΔNcPuf1 and iΔNcPuf1). A log-rank test was used to analyze significant differences between groups. ns = no significant difference, * 0.01 < *p* < 0.05, *** *p* < 0.001.

Plaque assay can fully reflect the vitality of *N. caninum* in vitro [22]. We first assessed the effect of ∆NcPuf1 on parasites by plaque assays. We found that the plaque size of ΔNcPuf1 strain was smaller than Nc-Liv (*p* < 0.05), and the plaque size was restored in the complementary strain (Figure 5A,B). The decrease in plaque size could have been caused by intracellular replication. We found that the replication of ΔNcPuf1 clones was slower than that of the Nc-Liv strain and the iΔNcPuf1 strain (*p* < 0.05) (Figure 5C).

To determine the influence of ΔNcPuf1 on the virulence in vivo, mice were infected with ΔNcPuf1, Nc-Liv, and iΔNcPuf1 tachyzoites by i.p. The results showed that all ΔNcPuf1-infected mice survived while the survival rate of the Nc-Liv-infected mice was 40%, and that of the iΔNcPuf1-infected mice was 40% (*p* < 0.05) (Figure 5D). Together, these results showed that NcPuf1 was vital for parasite growth in vitro and virulence in mice.

### 3.5. NcPuf1 Is Not Required for the Formation of P-Bodies in N. caninum

**Figure 6:**
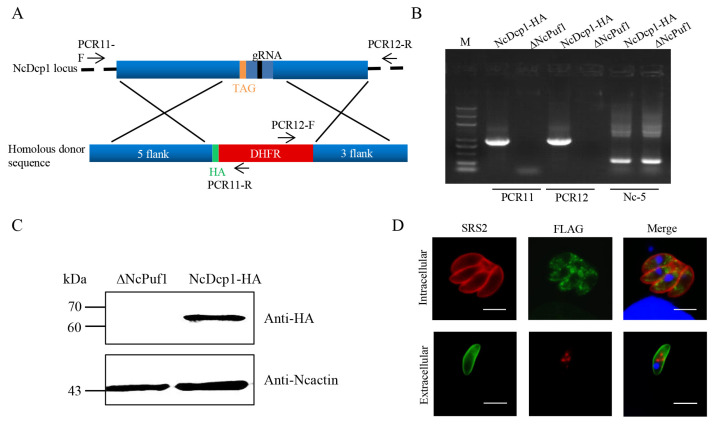
The localization of NcDcp1 in the NcPuf1 deficient strain. (**A**) Schematic representation of adding a HA epitope at the C-terminus of NcDcp1 in the parental ΔNcPuf1 strain. PCR11/ PCR12-F/R represent primers used to identify the recombination of the NcDcp1-HA-tagged strains. (**B**) The NcDcp1-HA-tagged strains were identified using PCR. PCR1 and PCR2 were used to identify the 5′ and 3′ insertions of HA-DHFR, respectively. The Nc-5 gene is a positive control gene. (**C**) Western blot analysis showed that *N. caninum* expressed decapping enzyme 1 (Dcp1) in the parental ΔNcPuf1 strain. (**D**) The HA-tagged NcDcp1 protein presented a granular cytoplasmic staining pattern in intracellular ΔNcPuf1 strain. Bars, 5 μm.

It is reported that the RNP granule would affect the viability of the extracellular parasites. Since NcPuf1 was located in P-bodies in the extracellular parasites, we wanted to know whether the deletion of NcPuf1 would affect the formation of P-bodies. The ΔNcPuf1 strain was co-transfected with the pSAG1::CAS9-NcU6::sgNcDcp1 and the linearized pLIC-HA-DHFR-NcDcp1(Figure 6A). The NcDcp1-HA-tagged strains were screened using pyrimethamine. The transfected parasites were recombined correctly by PCR (Figure 6B). The ΔNcPuf1 strain expressed the NcDcp1 protein by western blotting using mouse anti-HA antibody (Figure 6C). Then, we found that ΔNcPuf1 did not affect the formation of P-bodies by IFA using mouse anti-HA antibody (Figure 6D). This showed that NcPuf1 was not required for the formation of P-bodies in *N. caninum*.

### 3.6. ΔNcPuf1 does Not Affect the Differentiation from Tachyzoites to Bradyzoites In Vitro and Tissue Cysts Formation In Vivo

**Figure 7:**
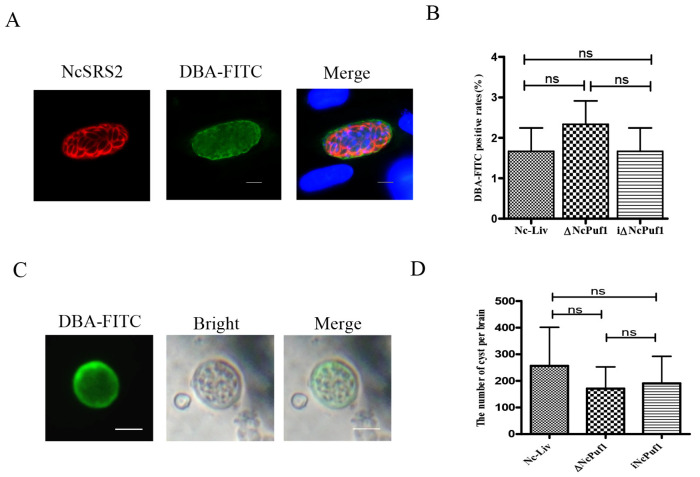
The effect of the NcPufs genes on the differentiation in vitro and tissue cysts formation in the mouse brain. (**A**) Cysts were detected by using FITC-labeled Dolichos biflorus agglutinin (DBA-FITC). The differentiation was induced under differentiation conditions (pH 8.2, low CO_2_) for 4 d. DBA-FITC-positive vacuoles are cysts. The scale bar represents 10 μm. (**B**) The positive staining for DBA-FITC vacuoles in different strains (Nc-Liv, ΔNcPuf1 and i ΔNcPuf1) were quantified by counting 100 parasitophorous vacuoles selected at random under a fluorescence microscope. (**C**) Tissue cysts from mouse brains were observed by IFA using DBA-FITC. (**D**)Tissue cysts were counted by IFA using DBA-FITC. Data are analyzed with One-way ANOVA with Tukey. ns = no significant difference.

It is generally believed that the cyst formation is related to a slowing of parasite growth. Therefore, we examined the effect of ΔNcPuf1 on the formation of bradyzoites. To determine the role of NcPuf1 in the differentiation from tachyzoites to bradyzoites in vitro, the Nc-Liv, ΔNcPuf1 and iΔNcPuf1 strains were induced to form cysts in vitro under alkaline conditions. Cyst walls were detected by using FITC-labeled Dolichos biflorus agglutinin (DBA-FITC) (Figure 7A). Unlike *T. gondii*, the bradyzoites of *N. caninum* has been difficult to obtain in vitro [23]. The parasites were cultured under the differentiation condition for 4 d in vitro. We found that ΔNcPuf1 and iΔNcPuf1 showed very similar DBA-FITC-positive rates to the parental Nc-Liv strain (*p* > 0.05) (Figure 7B).

To assess the effect of ΔNcPuf1 on the tissue cyst formation in vivo, we counted the number of brain cysts by DBA-FITC staining (Figure 7C). The Nc-Liv strain produced few cysts in the BALB/c mouse brain (data not shown), while more cysts were detected in Kunming mice. Thus, the brain cyst assay was performed by Kunming mice. The number of tissue cysts in mice infected with the ΔNcPuf1 strain was not reduced compared with that in Nc-Liv and iΔNcPuf1-infected mice brains (*p* > 0.05) (Figure 7D). We found that ΔNcPuf1 did not affect the formation of cysts in the mouse brains. Together, these results showed ΔNcPuf1 did not affect the differentiation in vitro and tissue cysts formation in vivo.

## 4. Discussion

Puf proteins have been identified in many organisms, but the number of them is different. Three Pufs were characterized in *Plasmodium* [24]. Additionally, two Pufs were found in *T. gondii* [12]. Two putative Puf genes were identified in *N. caninum* based on the conserved Puf domain, indicating that they may have some overlapping functions in *N. caninum.* However, their fulllengths share limited homology, suggesting that they may play different roles in *N. caninum.*

According to the primary function of Puf proteins, they are mainly located within the cytoplasm. However, a few Puf proteins are located in the nucleus or nucleolus. For example, Puf7 is located in the nucleolus in *T. brucei* [25], and Puf6p is found in the nucleus in *S. cerevisiae*. Here, we have shown that *N. caninum* possesses two putative Puf gene (NcPuf1 and NcPuf2). As expected, NcPuf1 localizes to the cytoplasm in intracellular parasites.

RNP granules have been detected in many eukaryotes and play an important role in the post-transcriptional regulation of genes [26]. Puf proteins were also found in RNP granule, including both SGs and P-bodies. Several have shown that Puf proteins are located in P-bodies. For example, both *T. cruzi* PUF6 and *C. elegans* germline FBF-2 localize to P-bodies [27,28]. PUM1 and PUM2 are also enriched in P-bodies [29]. Puf1, 2, and 3 are located in P-bodies under glucose deprivation in *Schizosaccharomyces pombe* [30]. Pufs are also found in SGs. Mammalian Pum2 is recruited to the SGs of hippocampal neurons during stress [31]. RNP granules, as a response to starvation, were reported in *T. cruzi* [32]. These indicated that Puf proteins might be localized in RNP granule when the cells were cultured under stress. Compared with intracellular parasites, extracellular parasites would be exposed to more stressful conditions. As expected, NcPuf1 forms punctate cytoplasmic structures in extracellular parasites. Finally, we found that NcPuf1 was located in P-bodies in extracellular parasites. *Saccharomyces cerevisiae* Puf3 can stimulate deadenylation of target mRNA by recruiting CNOT and Dhh1p [33]. For example, Puf3p promotes the degradation of COX17 mRNA in yeas [34]. Yeast Puf can bind Pop2p and subsequently recruited Ccr4p to the target mRNA to control deadenylation [35]. These suggest that Puf can bind the CCR4-NOT complex, a potentially conserved mechanism for target mRNA deadenylation. SGs and P-bodies contain many RBPs, including Puf proteins. P-bodies have many mRNAs and mRNA decay factors. Therefore, the result indicates that NcPuf1 may be involved in the degradation of the targeted mRNAs in extracellular parasites.

The knockout of NcPuf1 did not affect the formation of P-bodies. This result is similar to the finding that the deletion of the *Schizosaccharomyces pombe* puf1, 2, 3, and 4 genes did not affect the formation of P-bodies [30]. However, it differs from several reports that the loss of Puf affects the formation of P-bodies and SGs. Yeast Pufs are SGs components and are required for SGs formation [30]. The loss of mammalian Pum2 affects the formation of SGs in hippocampal neurons. This shows that NcPuf1 is not needed for P-bodies in *N. caninum*. NcPuf1 may use P-bodies as a signaling hub to coordinate the stress response in extracellular parasites.

The disruption of Mammalian PUM1 decreases cell proliferation via the posttranscriptional suppression of Cdkn1b [36]. PUF-8 promotes germline stem cell proliferation in *C. elegans* [37]. PUM2 participates in the positive control of adipose-derived stem cell proliferation [38]. While the PUM1 gene was silenced, the cell proliferation was significantly inhibited [39]. The deletion of PUM1 can decrease the proliferation of MIA, PaCa-2, and PANC-1 cells and promote their apoptosis [40]. Therefore, we speculate that the conserved function of the Puf protein is to promote proliferation. In this study, we found that NcPuf1 was critical for parasite growth in vitro. This shows that the functions of NcPuf1 conform to the conserved role of the Puf protein in promoting parasite proliferation.

Malaria gametocyte and sporozoite stages express two Puf proteins. The depletion of Puf1 does not result in a noticeable phenotype, whereas Puf2 plays vital roles in both the gametocyte and sporozoite stages. The deletion of PfPuf2 (ΔPfPuf2) or PbPuf2 (ΔPbPuf2) promotes the differentiation of gametocytes [41,42]. Transmission from mosquitoes to humans is also affected by Puf2 [11]. These results show that Puf is involved in the repression of differentiation. However, we found that NcPuf1 did not affect the differentiation from tachyzoites to bradyzoites in vitro and tissue cysts formation in vivo. FBF-1 and FBF-2 have opposite functions in the differentiation of *C. elegans* germline stem cells [43]. NcPuf2 may play an important role in differentiation. So the function of NcPuf2 needs to be further studied. In summary, we found that NcPuf1 plays a key role in tachyzoite proliferation in vitro and virulence in vivo, suggesting the potential of NcPuf1 as a vaccine/drug candidate against *N. caninum*.

## 5. Conclusions

In summary, we show that *N. caninum* encodes a Puf protein (NcPuf1). NcPuf1 is detected in the cytoplasm in intracellular parasites and in P-bodies, which are reported for the first time in *N. caninum* in extracellular parasites. NcPuf1 is not required for the formation of P-bodies in *N. caninum*. We find that ΔNcPuf1 does not affect the differentiation in vitro and tissue cysts formation in the mouse brain. However, ΔNcPuf1 significantly impairs tachyzoite replication in vitro and virulence in mice.

## Supplementary Materials

The following are available online at https://www.mdpi.com/2076-0817/9/12/1019/s1, Figure S1: NcPuf1 was not co-localized with Poly-A(+) mRNA binding protein(PABP), Table S1: The primer sequences used in this study.
