# Supplementary material for: NcPuf1 Is a Key Virulence Factor in Neospora caninum
Source: Pathogens. 2020 Dec 2;9(12):1019. doi: 10.3390/pathogens9121019 (PMC7761618; doi:10.3390/pathogens9121019)
Supplement: Supplementary file 1 [file pathogens-09-01019-s001.zip › Table S1.doc]

**Table S1. The primer sequences used in this study**

| **Primer name** | **Sequences** |
| --- | --- |
| 1 | ATACGACTCACTATAGGGCG |
| 2 | GCTCCACCGCGGTGGCGGCC |
| 3 | GCCGCCACCGCGGTGGAGCT |
| 4 | TCGTTCAACCCTCCCGCCGCAAACAACAATGTCCCTTTG |
| 5 | GCGGCGGGAGGGTTGAACGAGTTTTAGAGCTAGAAATAG |
| 6 | TCGCCCTATAGTGAGTCGTA |
| 7 | CTCGGTCAGTGCGAGACGAA |
| 8 | GCAGTTCGCCTCGACGCAGCTAT |
| 9 | AGTCCGGCAGTGACTGCTGT |
| 10 | CGAACTCGACAACCCAGAAG |
| 11 | TTCTGGGTTGTCGAGTTCGTCTAGAGCGGCCGCCACCGC |
| 12 | TCGTCTCGCACTGACCGAGGATATCTTAATTAAattgga |
| 13 | CTGCGTCGAGGCGAACTGCCCTAGGTACCCGTACGACGT |
| 14 | CAGCAGTCACTGCCGGACTACTAGTGGATCGATCCCCCG |
| 15 | CAGGAAACAGCTATGACC |
| 16 | TGTAAAACGACGGCCAGT |
| 17 | CAGAGCACACAGGTGCAAAT |
| 18 | atgacacacaggaactacgc |
| 19 | GGACGAATCCAGATGGAGAT |
| 20 | AAACCTACAGCACCACACAG |
| 21 | AAAGAGAAACGACCGGTCGCGTTTTAGAGCTAGAAATAG |
| 22 | GCGACCGGTCGTTTCTCTTTAAACAACAATGTCCCTTTG |
| 23 | ccaatTTAATTAAGATATCCTCTCGTCTCTCTCATCTCT |
| 24 | AGTCacttccacctccaccTCCACGCACATCCTCGCGA |
| 25 | CCGAGGGGGATCCACTAGAGACAGGAGACAGAAGAGACG |
| 26 | CGGTGGCGGCCGCTCTAGAGAATACGAGAGAGACTAGT |
| 27 | TCTAGAGCGGCCGCCACCGC |
| 28 | GATATCTTAATTAAattgga |
| 29 | ggtggaggtggaagtGACTA |
| 30 | TCTAGTGGATCCCCCTCGGG |
| 31 | TCTGACCGCTGCACATCTGG |
| 32 | GTGGGCAGACAGCAACAGTC |
| 33 | ACACAAGACAGCTATCAGTT |
| 34 | AATACGACAGAGACTAGAGA |
| 35 | GTGCGCTAAGGGCATCGGGTGTTTTAGAGCTAGAAATAG |
| 36 | ACCCGATGCCCTTAGCGCACAAACAACAATGTCCCTTTG |
| 37 | TCCGGAGTCTCCCCCGTCCC |
| 38 | GGTTGCGCCGACGCGCGAGA |
| 39 | AAAGGTCTCAGCGAAGGCGT |
| 40 | ATGCCAGCAGGCTCATCACA |
| 41 | CTCGCGCGTCGGCGCAACCTATAGGGCGAATTGGGTACC |
| 42 | CGCCTTCGCTGAGACCTTTGATATCTCTAGTGGATCCCC |
| 43 | GTGATGAGCCTGCTGGCATCCTAGGGCTAGCTCTAGAAC |
| 44 | GGACGGGGGAGACTCCGGAGCCGTCGTTTTACAACGTCG |
| 45 | AATCTGTCCAGTTCGCACG |
| 46 | TCACTCATTAGGCACCCCA |
| 47 | CCTCTATAGCCGTTTCCTCT |
| 48 | GCAACTGACTGAAATGCCTC |
| 49 | GGGGATCCACTAGAGATATC |
| 50 | ATCACTCACTCCATTCTGCC |
| 51 | GCTGCAGAGGATCTTGTGTT |
| 52 | AGCAATCTGTCGAACTGGCT |
| 53 | CAGTCAACCTACGTCTTCT |
| 54 | GTGCGTCCAATCCTGTAAC |
| 55 | GGTTTGCCGGCGCCTTCGTC |
| 56 | GGCGGAGCGTCGGGGGATG |
| 57 | GGCAATAAATACAAAGGCAACA |
| 58 | TTTCCAAACGGGTCTACG |
| 59 | CCTAGGTACCCGTACGACGT |
| 60 | GAATTCACTGGCCGTCGTTT |
| 61 | AACGACGGCCAGTGAATTCCTCTGACGGGGCTCTTCCTA |
| 62 | TTTGTCGAAAAAGGGAATTC |
| 63 | AATTCCCTTTTTCGACAAAATGAGTCTCAGCAGCCTCGA |
| 64 | CGTCGTACGGGTACCTAGGGCAGTTCGCCTCGACGCAGC |
| 65 | CCCCACGCTCTATCACGCGAG |
| 66 | ATGACACACAGGAACTACGC |
| 67 | TGGTATGTACGCAATAGCCC |
| 68 | GTTGTTCTGCTAACTCGTGG |
| 69 | TGCAAGTGCAAGATGGGGAT |
| 70 | ACTTCATCCTACACGCCTCA |
| 71 | CTCTCGTCTCTCTCATCTCT |
| 72 | TCCACGCACATCCTCGCGAC |
| 73 | GACAGGAGACAGAAGAGACG |
| 74 | GAATACGAGAGAGACTAGTG |
| 75 | ACTAGTCTCTCTCGTATTCTCTAGAGCGGCCGCCACCGC |
| 76 | GAGATGAGAGAGACGAGAGGATATCTTAATTAAattgga |
| 77 | TCGCGAGGATGTGCGTGGACCTAGGTACCCGTACGACGT |
| 78 | GTCTCTTCTGTCTCCTGTCACTAGTGGATCGATCCCCCG |
| 79 | CTTCTCGTACTATGGCCGGT |
| 80 | ccactcgtgaatgcgttat |
