# Supplementary material for: NcPuf1 Is a Key Virulence Factor in Neospora caninum
Source: Pathogens. 2020 Dec 2;9(12):1019. doi: 10.3390/pathogens9121019 (PMC7761618; doi:10.3390/pathogens9121019)
Supplement: Supplementary file 1 [file pathogens-09-01019-s001.zip › Figure S1.doc]

**Figure S1.** NcPuf1 was not co-localized with Poly-A(+) mRNA binding protein(PABP). IFA showed that HA-tagged NcPuf1 protein not co-localized with PABP protein that is the conserved marker of SGs in extracellular parasites. The scale bar represents 2 μm.
